# Investigation of 5-HT2B receptor induced dural plasma protein extravasation in a mouse migraine model

**DOI:** 10.1186/1129-2377-14-S1-P77

**Published:** 2013-02-21

**Authors:** A Hunfeld, D Segelcke, M Andriske, F Paris, X Zhu, H Lübbert

**Affiliations:** 1 Ruhr-Universität Bochum, Germany

## 

Migraine attacks originate in the meninges which are densely innervated by trigeminal nerve fibers. Stimulated endothelial cells of dural blood vessels secrete nitric oxide, where neuropeptides are released from trigeminal nerve fibers. This, in turn, leads to meningeal Plasma Protein Extravasation (PPE), serving as an established indicator for migraine attacks in animal models. In our mouse migraine model, we sensitized mice against the 5-HT2B receptor agonist meta-chlorphenylpiperazine (mCPP). Mice kept under hypoxic conditions for four weeks displayed significantly elevated PPEs in the dura mater upon mCPP injection. Tissue accumulation of the tracer Evans Blue was measured to quantify the extent of the PPE. In this study, several histological tracers were employed to identify the part of the vasculature where PPE occurs and the cellular mechanism associated with it. Injections of BSA-FITC (FITC-linked bovine serum albumin) verified the mCPP induced PPE in the dura mater. After leaving the blood vessels, the tracer was incorporated into perivascular, CD68-positive macrophages. With electron microscopic studies using the tracer HRP (horse radish peroxidase) we demonstrated that the PPE is associated with increased transcytotic transport. After 5-HT2B receptor activation, HRP escapes from capillaries and venules of hypoxic mice via an increased transcytotic transport in the endothelium. The hypoxic treatment itself increases transcytosis in arterioles, which may be indicative of a proinflammatory state of the endothelium. HRP was also detectable in intercellular clefts, but was always retained at tight junctions In summary, we demonstrated that in mice hypoxic treatment induces dural PPE via increased transcytotis at arterioles and that this is elevated to capillaries and venules after mCPP-injection.

